# 

**Published:** 1868-03

**Authors:** 


### BIBLIOGRAPHICAL NOTICES.

---

THE CHEMICAL NEWS.—This valuable scientific journal is now reproduced in this country at a very moderate expense. The London edition costs subscribers twelve dollars a year, while the American reprint is furnished at three dollars, and makes its appearance in monthly instead of weekly parts. Of the character of the publication it is not necessary to speak. It is too well known to need any commendation at this late day. Few men of science care to be without it, and it is invaluable to all who would keep up with the progress of Chemical and Physical Science.

W. A. Townsend & Adams, 434 Broome St., New York, are the publishers.

---

THE NEW ORLEANS JOURNAL OF MEDICINE.—The "New Orleans Medical and Surgical Journal" and the "Southern Journal of the Medical Sciences" have been fused into one under the title which heads this notice. The new journal is under the editorial management of Drs. S. M. Bemiss & W. S. Mitchell. It is a quarterly journal, well gotten up both editorially and mechanically. The original articles are numerous and valuable, and the selected matter is chosen with great judgment and embodies the most recent results in the medical sciences. We cordially welcome our new cotemporary and hope that its editors may meet with that abundant success they so richly deserve.

